# Genomic Prediction Can Provide Precise Estimates of the Genotypic Value of Barley Lines Evaluated in Unreplicated Trials

**DOI:** 10.3389/fpls.2022.735256

**Published:** 2022-04-22

**Authors:** Jérôme Terraillon, Matthias Frisch, K. Christin Falke, Heidi Jaiser, Monika Spiller, László Cselényi, Kerstin Krumnacker, Susanna Boxberger, Antje Habekuß, Doris Kopahnke, Albrecht Serfling, Frank Ordon, Carola Zenke-Philippi

**Affiliations:** ^1^Institute of Agronomy and Plant Breeding II, Justus Liebig University, Gießen, Germany; ^2^Saatzucht Josef Breun GmbH & Co. KG, Herzogenaurach, Germany; ^3^KWS Lochow GmbH, Northeim, Germany; ^4^W. von Borries-Eckendorf GmbH & Co. KG, Leopoldshöhe, Germany; ^5^Limagrain GmbH, Peine-Rosenthal, Germany; ^6^Ackermann Saatzucht GmbH & Co. KG, Irlbach, Germany; ^7^Institute for Resistance Research and Stress Tolerance, Julius Kühn Institute, Quedlinburg, Germany

**Keywords:** genomic prediction, barley, unreplicated trials, prediction accuracy, simulation

## Abstract

Genomic prediction has been established in breeding programs to predict the genotypic values of selection candidates without phenotypic data. First results in wheat showed that genomic predictions can also prove useful to select among material for which phenotypic data are available. In such a scenario, the selection candidates are evaluated with low intensity in the field. Genome-wide effects are estimated from the field data and are then used to predict the genotypic values of the selection candidates. The objectives of our simulation study were to investigate the correlations *r*(*y, g*) between genomic predictions *y* and genotypic values *g* and to compare these with the correlations *r*(*p, g*) between phenotypic values *p* and genotypic values *g*. We used data from a yield trial of 250 barley lines to estimate variance components and genome-wide effects. These parameters were used as basis for simulations. The simulations included multiple crossing schemes, population sizes, and varying sizes of the components of the masking variance. The genotypic values *g* of the selection candidates were obtained by genetic simulations, the phenotypic values *p* by simulating evaluation in the field, and the genomic predictions *y* by RR-BLUP effect estimation from the phenotypic values. The correlations *r*(*y, g*) were greater than the correlations *r*(*p, g*) for all investigated scenarios. We conclude that using genomic predictions for selection among candidates tested with low intensity in the field can proof useful for increasing the efficiency of barley breeding programs.

## 1. Introduction

Genomic selection between candidate genotypes that were not tested in field trials has been implemented successfully in breeding programs of major crops (Albrecht et al., 2011; Hofheinz et al., 2012; Auinger et al., 2016; Bartholomé et al., 2016). Genomic selection is carried out using either within-cyle prediction or across-cycle prediction. In within-cycle prediction, the selection candidates are split in two sets. The first set is evaluated in the field and used as a training set to estimate genomic effects which are then used to predict the performance of the second set of selection candidates. In across-cycle prediction, genotypes from the previous breeding cycle are used to predict the performance of selection candidates. Both applications focus on the prediction of genotypic values of selection candidates which were not yet evaluated in field trials.

Genomic predictions *y* can not only be employed to predict the genotypic values *g* of untested breeding material but also to predict the genotypic values *g* of tested material. This was suggested for preliminary yield trials in wheat by Endelman et al. (2014) and applied by Michel et al. (2017) and Michel et al. (2019). In such an approach, selection candidates are evaluated in the field with low testing intensity, typically one single plot per genotype. The phenotypic values *p* from the field trial are then used to estimate genomic marker effects. Subsequently, the genomic marker effects are used to calculate genomic predictions *y* of the true genotypic values *g*.

From a statistical point of view, this procedure is in analogy to the following example from linear regression. Assume for a given set of *x* values that the corresponding *y* values were assessed in an experiment. Linear regression puts a straight line ŷ through the scatterplot of the *x* and *y* values. To predict the *y* value of a certain *x* value we now use the predicted values ŷ on the regression line instead of the observed *y* values of the scatterplot. From a selection theory point of view, selection among tested selection candidates on basis of the genomic predictions *y* is preferable over selection on basis of phenotypic values *p* if the correlation *r*(*y, g*) is greater than the correlation *r*(*p, g*). To our knowledge there are no studies in barley that are investigating the use of genomic predictions to select between selection candidates that were evaluated in field trials.

The goal of our study was to investigate the correlations *r*(*p, g*) and *r*(*y, g*) with simulations based on an experimental barley data set. In particular, our objectives were to (i) estimate the correlations *r*(*p, g*) and *r*(*y, g*) for replicated and unreplicated trials based on simulations, (ii) investigate the effect of the mating scheme, population size, and the components of the masking variance on the correlations, and (iii) demonstrate that genomic selection among barley lines in unreplicated trials can be superior to phenotypic selection based on replicated trials.

## 2. Materials and Methods

### 2.1. Experimental Data Set

We used an experimental data set of winter barley (Osthushenrich et al., 2018) for obtaining estimates of genomic marker effects, genotypic values, and variance components. In our simulations, we assumed that these estimates were the true values of the respective parameters.

The experimental data set consisted of 250 doubled-haploid (DH) lines which were derived from 25 crosses of 10 parental lines. Five of the parental lines were elite lines and five were resistant donors. The derived lines and the parental lines were evaluated for yield at five locations in two years. The field trial was layed out as an augmented design with five blocks. In each block, 50 of the derived lines were tested together with the 10 parental lines that served as checks. In the first year, each of the derived lines was tested unreplicated in four locations, in the second year each of the derived lines was grown in two replications at five locations.

To estimate the adjusted treatment means of the of the parental lines and the derived lines we used the linear model


(1) $$\begin{array}{r} p=\mu +l+e+l:e+r:e+b:r:e+\varepsilon \end{array}$$

where *l* is the effect of the line, *e* is the effect of the environment, *l*:*e* is the genotype-by-environment interaction, *r*:*e* is the replication within environment effect, *b*:*r*:*e* is the block effect nested within replication and environment, and ε is the residual. The genotype was analyzed as a fixed factor, the remaining factors of the model were random. The adjusted treatment means ranged from 69.4 to 99.3 dt/ha (adjusted to 15% moisture), the standard errors of the means were 2.7 for the 10 check genotypes in the augmented design, and 3.1 for the 250 genotypes with one replication per block. The heritability for unbalanced trials, according to Equation (19) of Piepho and Möhring (2007), was *h*^2^ = 0.83.

To estimate the variance components from the derived lines we used the linear model


(2) $$\begin{array}{r} p=\mu +c+l:c+e+c:e+l:c:e+r:e+b:r:e+\varepsilon \end{array}$$

where *c* is the effect of a cross, *l*:*c* is the effect of the line within the cross, *e* is the environmental effect, *c*:*e* the cross-by-environment interaction, *l*:*c*:*e* the line-by-environment interaction, *r*:*e* is the replication within environment effect, *b*:*r*:*e* is the block effect nested within replication and environment, and ε is the residual. Assuming all effects as random, the cross-by-environment variance ${\hat{\sigma }}_{ce}^{2}=10.3$, the line-by-environment variance ${\hat{\sigma }}_{le}^{2}=11.1$, and the residual variance ${\hat{\sigma }}_{\varepsilon }^{2}=30.1$ were estimated with software ASReml-R (Butler et al., 2017).

The lines were genotyped with a 50 K single-nucleotide polymorphism (SNP) chip (Trait Genetics, Gatersleben). SNPs with more than two recorded alleles, more than 10% missing values and a gene diversity smaller than 0.1 were excluded from the analysis, as well as genotypes with more than 15% missing information. After preprocessing the marker data, 9,597 SNP markers and 259 genotypes (249 DH lines and 10 parental lines) remained for the analysis.

**Figure 1:**
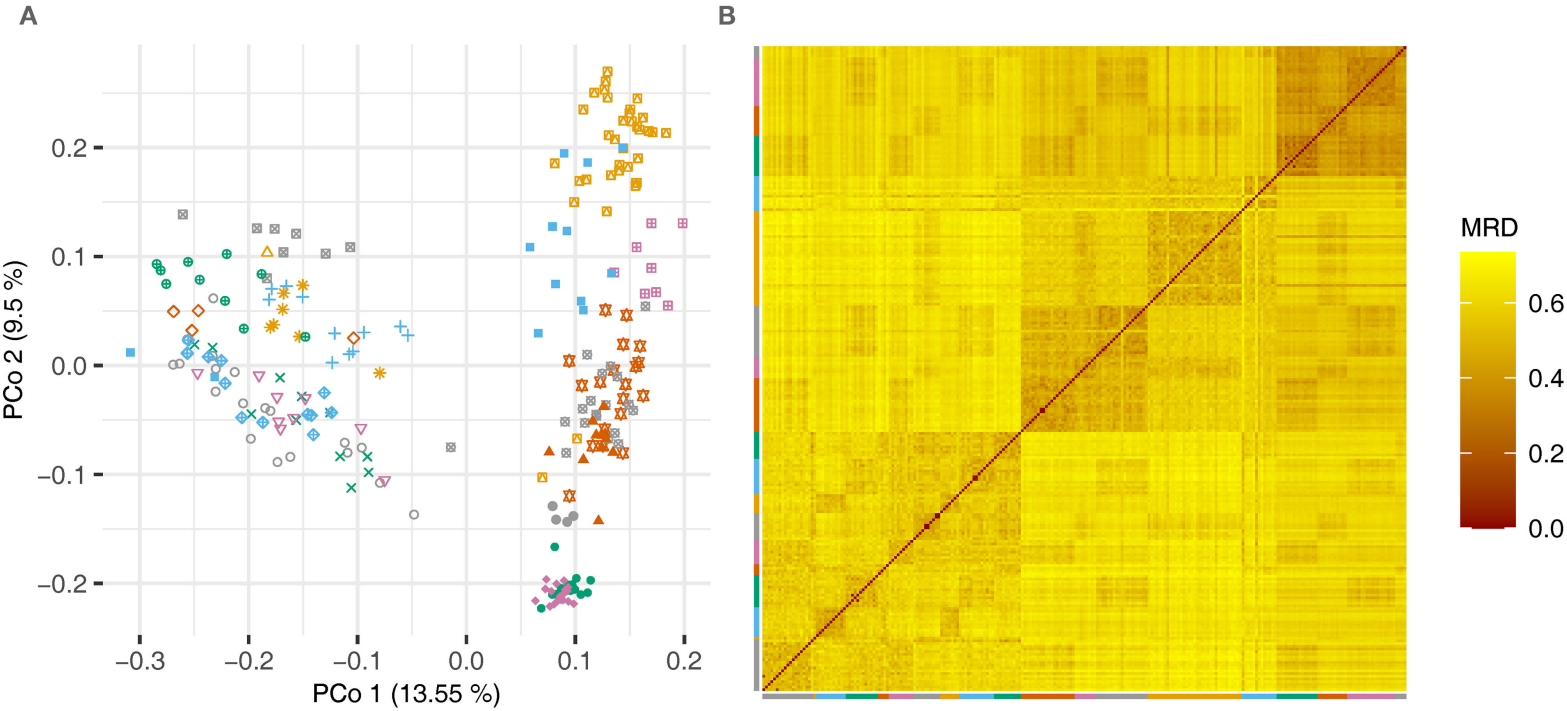
**(A)** Principal coordinate analysis based on the pairwise modified Roger's distances (MRD) and **(B)** heatmap showing the distances between the lines of the experimental barley data set. Lines that were derived from one cross are plotted using the same color and symbol.

The pairwise modified Roger's distances (*cf* Reif et al., 2005) between the lines were used in a principal coordinate analysis and a heatmap to illustrate the degree of relatedness of the lines (Figure 1).

From the adjusted treatment means, we estimated the genome-wide effects for yield with the RR-BLUP method (Meuwissen et al., 2001).

### 2.2. Simulation Methodology

Starting with the marker genotypes of the parental lines, we simulated the crosses and the development of DH lines from the crosses. The simulations were genetic simulations of recombination along chromosomes using the code of the software Plabsim (Maurer et al., 2008). The result of a simulation run are the marker genotypes of a set of simulated DH lines. The genotypic values *g* of the simulated DH lines were determined from their marker genotypes and the corresponding genomic marker effects. For this purpose, we used the genomic marker effects obtained from the experimental data set and assumed that these were the true values. This procedure results in simulated DH lines of which the marker genotypes as well as the genotypic values *g* are known. The genotypic value of the *l*-th line of the *c*-th cross is denoted by *g*_*cl*_.

The evaluation of the DH lines in field trials at *E* environments with *R* replications per environment was simulated using normally distributed random numbers. The masking effect for the *l*-th line of the *c*-th cross was *m*_*cl*_ = *u*_*c*_+*v*_*l*_+*w*_ε_ where *u*_*c*_, *v*_*l*_, and *w*_ε_ were realizations of independent random variables with distribution $u\sim N\left(0,\sigma_{ce}^{2}/E\right)$, $v\sim N\left(0,\sigma_{le}^{2}/E\right)$, and $w\sim N\left[0,\sigma_{\varepsilon }^{2}/\left(ER\right)\right]$. The variance components $\sigma_{ce}^{2}$, $\sigma_{le}^{2}$, and $\sigma_{\varepsilon }^{2}$ were determined on basis of the variance components estimated from the experimental barley data as described in the subsequent section on the simulated scenarios. The effects *u*_*c*_ were determined for each cross and the effects *v*_*l*_ for each DH line. With the simulated genotypic values *g*_*cl*_ and the masking effects *m*_*cl*_, the phenotypic values were determined as *p*_*cl*_ = *g*_*cl*_+*m*_*cl*_.

The phenotypic values *p*_*cl*_ of the simulated DH lines were in turn used to estimate simulated genomic marker effects with the RR-BLUP method. The simulated genomic marker effects were then used to calculate genomic predictions *y*_*cl*_ of the true genotypic values *g*_*cl*_.

### 2.3. Simulated Scenarios

In order to illustrate the principle of using genomic prediction for selection candidates evaluated in unreplicated field trials, we simulated two experiments with a mating scheme similar to that of Osthushenrich et al. (2018) as a starting point. These two experiments were simulated with only one simulation run. After a factorial cross of the 5 × 5 parental lines, 10 DH lines were generated from each cross, resulting in 250 DH lines.

We considered two different field trial designs for the evaluation of the 250 simulated DH lines in order to compare unreplicated and replicated trials. The first investigated design was an unreplicated trial with one environment (*E* = 1) and one plot per genotype (*R* = 1). The second investigated design was a replicated trial with three environments (*E* = 3) and two replications per environment (*R* = 2). The variance components $\sigma_{ce}^{2}$, $\sigma_{le}^{2}$, and $\sigma_{\varepsilon }^{2}$ that were estimated from the experimental data set were used for calculating the masking effects *m*_*cl*_ and thus the simulated phenotypic values *p*_*cl*_ for the two different field trial designs.

In order to investigate the effect of varying the variance components $\sigma_{ce}^{2}$, $\sigma_{le}^{2}$, and $\sigma_{\varepsilon }^{2}$ of the masking variance for unreplicated (*E* = 1, *R* = 1) and replicated (*E* = 3, *R* = 2) field trials, we ran a set of replicated simulations with 2,000 simulation runs for each parameter setting. In addition to the original factorial crossing scheme of 5 × 5 lines, we also simulated a diallel mating scheme. In the diallel mating scheme, all 10 pairwise crosses between the 5 elite lines were carried out.

In the factorial mating scheme we simulated either family sizes of 10 DH lines per cross, resulting in a population of 250 lines, or family sizes of six lines per cross, resulting in a population size of 150 lines. For the diallel mating scheme, we generated 25 DH lines for each of the 10 crosses, resulting in a population size of 250 lines. Thus, we investigated three different mating schemes, all of which were evaluated in both an unreplicated (*E* = 1, *R* = 1) and a replicated (*E* = 3, *R* = 2) field trial.

For each of these six scenarios, we considered five different sets of variance components $\sigma_{ce}^{2}$, $\sigma_{le}^{2}$, and $\sigma_{\varepsilon }^{2}$ of the masking variance in order to assess the effect on the correlations *r*(*p, g*), *r*(*y, p*), and *r*(*y, g*).

For the first set of variance components, we used a setting that is close to the variance components of the experimental design: $\sigma_{ce}^{2}=10,\sigma_{le}^{2}=10,\sigma_{\varepsilon }^{2}=30$. For the second set of variance components, we used a smaller cross-by-environment variance and a greater line-by-environment variance than in the experimental data set: $\sigma_{ce}^{2}=2,\sigma_{le}^{2}=18,\sigma_{\varepsilon }^{2}=30$. For the third set of variance components, we used a greater cross-by-environment variance and a smaller line-by-environment variance than in the experimental data set: $\sigma_{ce}^{2}=18,\sigma_{le}^{2}=2,\sigma_{\varepsilon }^{2}=30$. In addition, we investigated a scenario with a greater error variance: $\sigma_{ce}^{2}=10$, $\sigma_{le}^{2}=10$, $\sigma_{\varepsilon }^{2}=60$, and one in which the interaction variances as well as the error variance were considerably greater than in the experimental data set: $\sigma_{ce}^{2}=20$, $\sigma_{le}^{2}=20$, $\sigma_{\varepsilon }^{2}=120$.

In phenotypic selection, the phenotypic values *p* assessed in field trials are used as estimates for the genotypic values *g* of the selection candidates. The correlation *r*(*p, g*) depends on the heritability and determines the response to selection. The response to genomic selection is determined by the correlation *r*(*y, g*) between the genomic predictions *y* and the genotypic values *g*. The correlation *r*(*y, g*) is referred to as prediction accuracy (Legarra et al., 2008). We used the mean values of the correlations across the replicated simulations to compare the efficiency of phenotypic selection with genomic selection.

The R code used to carry out the simulations is available at Github (https://github.com/JT-Giessen/Terraillon_2022).

### 2.4. Retrospective Re-analysis of the Experimental Data

Our experimental data was collected in two years, in the first year one plot per genotype was planted in each of four locations (*R* = 1, *E* = 4), in the second year two replications were grown in each location (*R* = 2, *E* = 5). In the retrospective analysis we regarded the data from each of the four environments in year one as an unreplicated trial (*R* = 1, *E* = 1). This results in four sets of phenotypic values. We denote the sets of phenotypic values obtained from these unreplicated trials with p^*^.

We then used the four sets of phenotypic values p^*^ to estimate four sets of genomic effects using the RR-BLUP method. From these we calculated four sets of genomic performance estimates y^*^. For each set of values p^*^ we used the remaining three locations of the first year and the data from the second year to determine the adjusted treatment means of the lines according to Equation (1). These phenotypic values were then regarded as estimators for the genotypic values ĝ^*^.

For each of the four sets of values p^*^, y^*^, and ĝ^*^ we determined the correlations *r*(p^*^, ĝ^*^) between the phenotypic values and the estimate of the genotypic value, and *r*(y^*^, ĝ^*^) between the genomic prediction and the estimate of the genotypic value.

A one-sided Pearson *z*-test with the alternative hypothesis *H*_*A*_: *r*(p^*^, ĝ^*^) < *r*(y^*^, ĝ^*^) was carried out with the R package cocor (Diedenhofen and Musch, 2015) to test whether the genomic predictions are superior to the phenotypic values in predicting the estimated genotypic value.

## 3. Results

**Figure 2:**
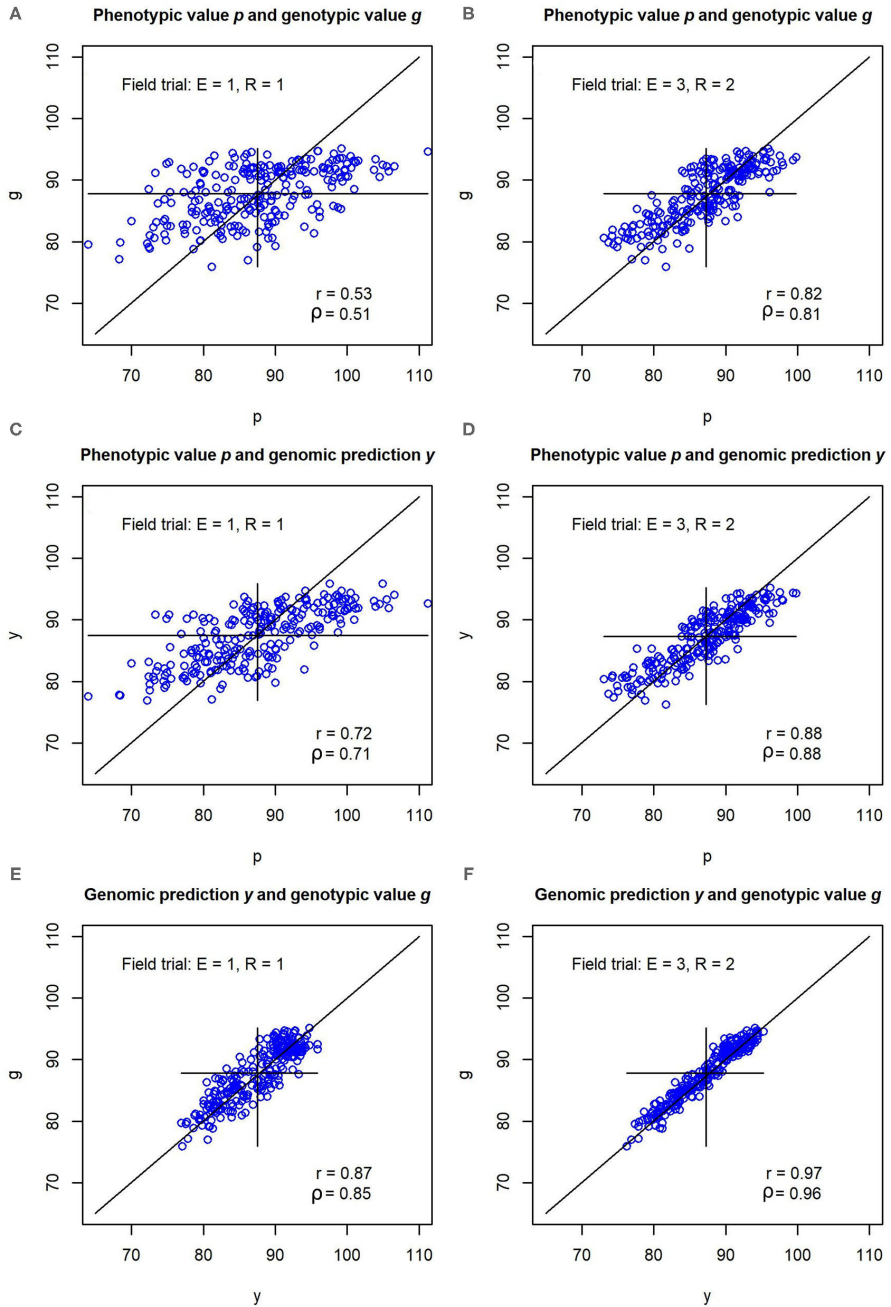
**(A–F)** Pearson correlations *r* and Spearmans correlations ρ between phenotypic values *p*, genomic predictions *y*, and genotypic values *g* for a simulated data set. The simulation was carried out with variance components and genomic effects estimated from an experimental barley data set. Unreplicated field evaluation (*E* = 1, *R* = 1) is compared with a replicated multi-environment trial (*E* = 3, *R* = 2).

In the illustration example that was based on the parameter settings and variance components from the experimental data set, the pairwise correlations *r*(*p, g*), *r*(*y, p*), and *r*(*y, g*) between phenotypic values *p*, genotypic values *g* and genomic predictions *y* were consistently lower for the unreplicated trial than for the replicated multi-environment trial (Figure 2). The correlations amounted to *r*(*p, g*) = 0.53, *r*(*y, p*) = 0.72, and *r*(*y, g*) = 0.87 in the unreplicated trial, and *r*(*p, g*) = 0.82, *r*(*y, p*) = 0.88, and *r*(*y, g*) = 0.97 in the replicated trial. The correlation *r*(*y, g*) in the unreplicated trial thus surpassed the correlation *r*(*p, g*) in the replicated trial, even though the unreplicated trial used only one sixth of the field plots of the replicated trial.

The replicated simulations, which investigated different mating schemes, population sizes and sets of variance components, confirmed the results of the illustration example with respect to the correlations *r*(*p, g*), *r*(*y, p*), and *r*(*y, p*) (Table 1). All correlations were lower for the unreplicated trials (l. 1–5, 11–15, 21–25 of Table 1) than for the replicated trials (l. 6–10, 16–20, 26–30 of Table 1). The difference between replicated and unreplicated trials was lowest for *r*(*y, g*) and highest for *r*(*p, g*). The correlations *r*(*p, g*) were consistently lower than both *r*(*y, p*) and *r*(*y, g*) for all investigated scenarios (Table 1). In the factorial mating designs, the correlations *r*(*p, g*) between phenotypic and genotypic values ranged from 0.33 to 0.54 for the unreplicated trials and from 0.61 to 0.80 for the replicated trials (l. 1–20 of Table 1). The correlations *r*(*y, g*) between genomic predictions and genotypic values ranged from 0.76 to 0.91 for the unreplicated trials and from 0.89 to 0.96 for the replicated trials (l. 1–20 of Table 1).

**Table 1:** Pearson correlations between the phenotypic values and the genotypic values *r*(*p, g*), between the genomic predictions and the phenotypic values *r*(*y, p*), and between the genomic predictions and the genotypic values *r*(*y, g*) depending on the mating scheme, the number of crosses *n*_*c*_, the number of lines per cross *n*_*l*_, the number of environments *E* and replications per environment *R*, the cross-by-environment variance $\sigma_{ce}^{2}$, the line-by-environment variance $\sigma_{le}^{2}$, the residual variance $\sigma_{\varepsilon }^{2}$, and the genetic variance $\sigma_{g}^{2}$.

	***n*_*c*_**	***n*_*l*_**	**E**	**R**	** $\sigma_{ce}^{2}$ **	** $\sigma_{le}^{2}$ **	** $\sigma_{\varepsilon }^{2}$ **	** $\sigma_{g}^{2}$ **	***r*(*p, g*)**	***r*(*y, p*)**	***r*(*y, g*)**
**Factorial mating**											
1	25	10	1	1	10	10	30	20	0.53	0.69	0.88
2	25	10	1	1	2	18	30	20	0.53	0.67	0.91
3	25	10	1	1	18	2	30	20	0.53	0.71	0.85
4	25	10	1	1	10	10	60	20	0.44	0.62	0.86
5	25	10	1	1	20	20	120	20	0.33	0.54	0.80
6	25	10	3	2	10	10	30	20	0.80	0.88	0.94
7	25	10	3	2	2	18	30	20	0.79	0.87	0.96
8	25	10	3	2	18	2	30	20	0.80	0.89	0.93
9	25	10	3	2	10	10	60	20	0.74	0.84	0.94
10	25	10	3	2	20	20	120	20	0.61	0.75	0.90
11	25	6	1	1	10	10	30	20	0.53	0.73	0.86
12	25	6	1	1	2	18	30	20	0.53	0.71	0.88
13	25	6	1	1	18	2	30	20	0.54	0.75	0.83
14	25	6	1	1	10	10	60	20	0.45	0.66	0.83
15	25	6	1	1	20	20	120	20	0.33	0.58	0.76
16	25	6	3	2	10	10	30	20	0.80	0.90	0.93
17	25	6	3	2	2	18	30	20	0.80	0.89	0.94
18	25	6	3	2	18	2	30	20	0.80	0.91	0.92
19	25	6	3	2	10	10	60	20	0.74	0.86	0.92
20	25	6	3	2	20	20	120	20	0.61	0.78	0.89
**Diallel mating**											
21	10	25	1	1	10	10	30	2	0.19	0.40	0.43
22	10	25	1	1	2	18	30	2	0.20	0.32	0.52
23	10	25	1	1	18	2	30	2	0.19	0.46	0.37
24	10	25	1	1	10	10	60	2	0.16	0.34	0.38
25	10	25	1	1	20	20	120	2	0.11	0.31	0.26
26	10	25	3	2	10	10	30	2	0.38	0.56	0.69
27	10	25	3	2	2	18	30	2	0.38	0.52	0.79
28	10	25	3	2	18	2	30	2	0.38	0.61	0.62
29	10	25	3	2	10	10	60	2	0.33	0.50	0.66
30	10	25	3	2	20	20	120	2	0.24	0.44	0.52

All correlations were of similar size for both investigated population sizes of the factorial mating scheme (l. 1–20 of Table 1). The superiority of the genomic predictions *y* over the phenotypic values *p* persisted when the total population size of the factorial mating designs was reduced from 250 to 150 DH lines (compare l. 1–10 to l. 11–20 of Table 1). In both factorial mating scenarios, the correlations *r*(*y, g*) in the unreplicated trials were higher than the correlations *r*(*p, g*) in the corresponding replicated trials (l. 1–20 of Table 1). However, the differences between *r*(*y, g*) in the unreplicated trials and *r*(*p, g*) in the replicated trials diminished when the total population size was reduced to 150 DH lines. For example, in the scenario that was close to the experimental data set, *r*(*y, g*) in the unreplicated trial was 0.88 and *r*(*p, g*) in the replicated trial was 0.80 with a population size of 250 DH lines (l. 1 and 6 of Table 1). With 150 DH lines, *r*(*y, g*) in the unreplicated trial diminished to 0.86, while *r*(*p, g*) in the replicated trial remained at 0.80 (l. 11 and 16 of Table 1).

The masking variance for the simulation of the phenotypic values *p* contained three different variance components: the cross-by-environment variance $\sigma_{ce}^{2}$, the line-by-environment variance $\sigma_{le}^{2}$, and the error variance $\sigma_{\varepsilon }^{2}$. We investigated five different sets of these variance components. For the first three sets, the error variance was held constant at $\sigma_{\varepsilon }^{2}=30$. In these scenarios, the correlation *r*(*y, g*) decreased with increasing $\sigma_{ce}^{2}$ from 2 to 10 to 18, while the correlation *r*(*y, p*) increased. For example, in the factorial mating scheme with 10 DH lines per cross, *r*(*y, g*) decreased from 0.91 to 0.88 to 0.85, while *r*(*y, p*) increased from 0.67 to 0.69 to 0.71 (l. 2, 1, and 3 of Table 1). Conversely, with increasing $\sigma_{le}^{2}$ from 2 to 10 to 18, the correlation *r*(*y, g*) increased, while the correlation *r*(*y, p*) decreased (l. 3, 1, and 2 of Table 1). Thus, the correlation *r*(*y, p*) was lowest and the correlation *r*(*y, g*) was highest for $\sigma_{ce}^{2}=2$ and $\sigma_{le}^{2}=18$ for all scenarios with $\sigma_{\varepsilon }^{2}=30$ (l. 2, 7, 12, 17, 22, and 27 of Table 1). The correlation *r*(*p, g*) remained approximately constant with increasing $\sigma_{ce}^{2}$ for all scenarios with $\sigma_{\varepsilon }^{2}=30$.

Increasing the error variance $\sigma_{\varepsilon }^{2}$ from 30 to 60 resulted in a reduction of all three correlation coefficients *r*(*p, g*), *r*(*y, p*), and *r*(*y, g*) for all investigated mating schemes (l. 1, 4, 6, 11, 14, 16, 19, 21, 24, 26, and 29 of Table 1). The only exception was the correlation *r*(*y, g*) in the factorial mating scheme with a population size of 250 in the replicated trials. It increased slightly when $\sigma_{\varepsilon }^{2}$ was increased from 30 to 60 (l. 9 of Table 1). Further increasing the error variance $\sigma_{\varepsilon }^{2}$ to 120 and both $\sigma_{ce}^{2}$ and $\sigma_{le}^{2}$ to 20 diminished the correlation coefficients even more (l. 5, 10, 15, 20, 25, and 30 of Table 1).

In addition to the factorial mating schemes, we also simulated two diallel mating schemes for the five elite parents. In the diallel mating schemes, the genetic variance was estimated at $\sigma_{g}^{2}=2$ (l. 21–30 of Table 1). This is a strong reduction in comparison to the genetic variance of the factorial mating schemes with a genetic variance of $\sigma_{g}^{2}=20$ (l. 1–20 of Table 1). The correlations *r*(*p, g*), *r*(*y, p*), and *r*(*y, g*) were consistently much lower in the diallel mating schemes than in the factorial mating schemes (l. 21–30 of Table 1). For example, *r*(*p, g*) ranged from 0.11 to 0.20 in the unreplicated trials and from 0.24 to 0.38 in the replicated trials (l. 21–30 of Table 1). The correlation *r*(*y, g*) ranged from 0.26 to 0.52 in the unreplicated trials and from 0.52 to 0.79 in the replicated trials (l. 21–30 of Table 1). However, as for the factorial mating schemes, *r*(*y, g*) in the unreplicated trials was higher than or comparable to *r*(*p, g*) in the corresponding replicated trials (l. 21–30 of Table 1). In contrast to the factorial mating schemes, for which *r*(*y, p*) was always lower than *r*(*y, g*), *r*(*y, p*) was higher than *r*(*y, g*) for a higher cross-by-environment/line-by-environment variance ratio and larger environmental variances in the unreplicated trials (l. 23 and 25 of Table 1).

In the retrospective re-analysis of the experimental data, the correlations *r*(y^*^, ĝ^*^) between genomic predictions and genotypic values were for all four sets of data greater than the correlations *r*(p^*^, ĝ^*^) between the phenotypic values from the unreplicated trial and the genotypic values (Table 2). In three out of four cases the superiority was significant (α = 0.05).

**Table 2:** Correlations *r*(p^*^, ĝ^*^) between the phenotypic values from an unreplicated trial and the genotypic values, correlations *r*(y^*^, ĝ^*^) between genomic predictions and the genotypic values, and *p*-value of the *z*-test to compare the two correlations for the four data sets of the retrospective re-analysis of the experimental data.

**Data set**	*r*(p^*^, ĝ^*^)	*r*(y^*^, ĝ^*^)	***p*-value**
1	0.57	0.62	0.0166
2	0.63	0.66	0.1568
3	0.62	0.66	0.0316
4	0.61	0.69	0.0019

## 4. Discussion

### 4.1. Modeling the Breeding Program

In our study, we use a model of a breeding program for our simulations. Here we discuss briefly the rationales behind choosing its components.

In a field trial, the environment, the genotype-by-environment interaction, the design factors, such as blocks or replications, and the experimental error contribute to the phenotypic value of the tested genotypes. This is modeled in our approach by adding a masking effect to the genotypic value. If, for a given genotype, *n* replications with respect to a given variance component σ^2^ are available, then averaging over the *n* replications results in a transformed random variable with variance σ^2^/*n*. Summing up realizations of transformed random variables for all variance components of a trial allows to model environmental conditions similar to those of the trial from which the variance components were estimated.

In applied breeding programs, it is a common approach to select the best lines available from one cycle of material development and to recombine these as parents to obtain the selection candidates for the next cycle. If the lines are from one material group, then one option for recombining the lines is to mate every line with every other line, this corresponds to a diallel mating scheme. If the lines originate from two pools, for example, a resistance pool and a pool with high yielding genotypes, then one option to recombine the lines is to mate every line from the first pool to every line of the second pool. This corresponds to a factorial mating scheme. This was the rationale for investigating both, the diallel and the factorial mating scheme in our study.

### 4.2. Simulation Methodology

In experimental evaluations of genomic prediction, the correlation *r*(*y, p*) between genomic predictions *y* and phenotypic values *p* is often used as a measure for the precision of prediction (e.g., Hofheinz et al., 2012; Albrecht et al., 2014; Lorenz and Smith, 2015; Zenke-Philippi et al., 2017; Werner et al., 2018). The reason for this is that the correlation *r*(*y, g*) between the genomic predictions *y* and the true genotypic values *g* remains unknown. Some authors divide the correlation *r*(*y, p*) by the square root of the heritability (e.g., Albrecht et al., 2011; Zhao et al., 2013; Technow et al., 2014; Sallam et al., 2015). This is a linear transformation, and the ranking of selection candidates remains the same as in the untransformed data assuming constant heritability. The simulation methodology employed in this study provides, in contrast to experimental evaluations, a direct assessment of the correlations *r*(*p, g*) and *r*(*y, g*) between the phenotypic values *p* and genotypic values *g* and between the genomic predictions *y* and the genotypic values *g*, respectively. This allows the comparison of the selection criteria *p* and *y* with respect to their precision to predict the true unknown genotypic values *g* of selection candidates. Moreover, it facilitates the investigation of quantitative genetic factors that affect the two correlations.

The genomic marker effects and the components of the masking variance from our experimental barley data set were used as the basis for the simulations. This methodology implies simplifying assumptions, the most important of which we point out here briefly. The purely additive genetic model neglecting epistasis as well as the estimation method for genomic marker effects might have an effect on the results. It is neglected that a certain SNP variant might be in linkage disequilibrium with alleles having different genomic effects in breeding material that originates from genetically different sources. Moreover, we assume that the genomic effects of SNPs were estimated in the original barley data set without a residual error. In particular this simplification might result in an overestimation of the correlations of *y* and *p* with the true genotypic values *g*. In addition, the genetic structure of the parental lines can be assumed to have an effect on the size of the estimated genomic marker effects.

We used genome-wide effects estimated from an experimental data set as the basis for our simulations. An alternative approach is to use genetic effects that were drawn with a random number generator from a probability distribution. A prominent example for this methodology is Meuwissen et al. (2001). We have chosen to use effects estimated from experimental data, because we think that this might be closer to reality than effects from a random number.

In consequence, the results reported here can only illustrate the concept of using genomic predictions of tested material. Further research is needed on the basis of other experimental data sets or quantitative genetic scenarios to confirm the transferability to other situations of the results reported here.

### 4.3. Illustration of the Principle of Using Genomic Predictions of Tested Material

In the simulation using the variance components and genomic marker effects of Osthushenrich et al. (2018), the correlation *r*(*p, g*) between the phenotypic values *p* and the genotypic values *g* in an unreplicated field trial was 0.53 (Figure 2). The strength of this correlation might just be sufficient for selection, but the selection gain that can be reached is low.

In a field trial in three environments with two replications (*E* = 3, *R* = 2), the correlation between the phenotypic and the genotypic values amounted to *r*(*p, g*) = 0.82, which should allow for efficient selection with reasonable selection gain. However, the increase in precision in comparison to the unreplicated field trial comes at the cost of a six-fold increase of the resources required for the field trial.

In contrast, the correlation *r*(*y, g*) between the genomic predictions and the true genotypic values amounted to *r*(*y, g*) = 0.87 in the unreplicated trial (Figure 2). Hence, the marker-based genomic predictions *y* of the genotypic value have a much higher correlation with the true genotypic value *g* than the phenotypic values *p* of the unreplicated trial. The correlation reaches values that are typically reached by replicated trials and even surpasses the correlation *r*(*p, g*) reached by the replicated field trial with *E* = 3, *R* = 2. This enables an efficient selection of genotypes with high performance which results in a high selection gain.

From an applied point of view, the costs of genotyping a line might equal roughly the costs of one field plot. In consequence, our results suggest for the investigated data set that using one field plot and in addition genotyping the lines can realize a selection gain roughly corresponding to that of a replicated trial, but requires only a third of the resources.

### 4.4. Superiority of Genomic Predictions Over Phenotypic Estimates

The correlations *r*(*y, g*) for unreplicated trials reached values that surpassed the correlations *r*(*p, g*) for the corresponding replicated trials for all investigated scenarios (Table 1). For example, an experimental design close to the experimental data set resulted in the correlation *r*(*y, g*) = 0.88 for the unreplicated trial (l. 1 of Table 1) and in the correlation *r*(*p, g*) = 0.80 for the replicated trial (l. 6 of Table 1). We conclude that using unreplicated trials combined with genomic selection might be a useful strategy for a wide range of genetic situations, which we address subsequently.

The lower limit for the population size that is required to apply the proposed approach of predicting tested lines is not yet reached with 150 individuals. This conclusion is supported by the *r*(*y, g*) = 0.86 of l. 11 of Table 1 and *r*(*p, g*) = 0.80 presented in l. 16 of Table 1.

In our analysis we divided the genotype-by-environment interaction variance into two components, the cross-by-environment variance $\sigma_{ce}^{2}$ and the line-by-environment variance $\sigma_{le}^{2}$. With an increasing importance of the cross-by-environment component of $\sigma_{ce}^{2}=2,10,18$ (l. 2, 1, 3 of Table 1), the correlation between genomic predictions and genotypic values *r*(*y, g*) decreased while the correlation *r*(*y, p*) increased. This result suggests that a large genotype-by-cross variance might render the assessment of prediction accuracy using the correlation *r*(*y, p*) difficult as there might be situations in which changing parameters of a genomic selection program increases *r*(*y, p*) without actually increasing *r*(*y, g*).

With increasing error variances and increasing genotype-by-environment variances (e.g., l. 1, 4, 5 of Table 1), the correlations *r*(*p, g*) decreased considerably. The correlations *r*(*y, g*) remained above 0.75 nevertheless in the factorial mating schemes. This suggests that even when the correlations between the phenotypic and genotypic values of an unreplicated trial get low, using genomic predictions instead can still enable successful genomic selection of candidate genotypes.

In the diallel mating scheme of the elite lines, the genetic variance was only a fraction of the genetic variance in the complete factorial. This observation might be caused by the contrasting yield of the two parental groups in the factorial mating scheme. The small genetic variance in comparison to the components of the masking variance in the diallel mating scheme results in a low heritability in the unreplicated trial accompanied by a correlation *r*(*p, g*) of 0.19 or 0.20 (l. 21–23 of Table 1). Even in this extreme scenario, using the genomic predictions for the genotypic values for selection results in correlations *r*(*y, g*) of 0.37 to 0.52.

These comparisons suggest that using genomic predictions *y* of the genotypic values might be preferable over using the phenotypic values *p* directly for a wide range of variance components and populations sizes with our data set.

### 4.5. Pseudo-Replications of Genome Stretches

The high correlation between the true genotypic values *g* and their genomic predictions *y* observed in this study might be explained by the replicated evaluation of chromosome segments in different lines. Due to the replicated use of the parental lines, the parental chromosome segments are present and evaluated in the field in a large number of derived lines. The amount of this pseudo-replication of chromosome segments strongly depends on the degree of relatedness between the evaluated lines and the crossing scheme that was used to develop them.

From a quantitative genetics point of view, the approach suggested here can be related to older best linear unbiased prediction (BLUP) approaches. Assuming a large number of markers with small genomic effects of similar size, the genomic prediction model converges to a GBLUP model that employs the realized relationship matrix between the tested genotypes. Those type of prediction models showed a good performance in maize (Bernardo, 1994). A direct consequence of these hypotheses on the mechanism behind the observed results is that the approach suggested here can only be expected to work if the material under investigation is related. This, however, is typically the case in breeding programs for cultivar development, because often several selection candidates were derived from the same crosses, and the parents of the crosses are used in several crosses.

### 4.6. Retrospective Re-analysis of the Experimental Data Set

A thorough experimental validation of the results obtained from the presented simulation study requires an experimental data set that estimates the genotypic values of the tested lines with high precision. To achieve highly precise estimates, typically trials over several years (e.g., more than tree) at many locations (e.g., more than 10) are required. The precision of the field trial used to collect our experimental data can be considered as sufficient in applied breeding programs, but it does not provide highly precise estimates of the genotypic value. In consequence, this data set can not be used for a rigid experimental validation of our simulation results. Nevertheless, it can be used to demonstrate the potential usefulness of our approach.

In the retrospective re-analysis we estimated the genotypic values ĝ^*^ from three locations with one plot per genotype in the first year and five locations with two replications per genotype in the second year. The fourth location in the first year was considered as a “preliminary yield trial” (in terms of Endelman et al., 2014) to obtain the phenotypic values p^*^. The research question was, whether the estimations of the genotypic values ĝ^*^ can be better approximated by the field data p^*^ of single-plot experiments or by genomic predictions y^*^ obtained from the field data of single-plot experiments.

For all four data sets, which correspond to the four single-plot experiments in the first year, we observed a greater correlation *r*(y^*^, ĝ^*^) than *r*(p^*^, ĝ^*^). Hence, the genotypic predictions from single-plot experiments approximated the genotypic values better than the original phenotypic values of the experiments.

## 5. Conclusion

Our investigation focused on the research question whether genomic selection between genotypes tested with low intensity in a field trial can be superior to phenotypic selection. We conclude, that for breeding material where parental lines are used in several crosses and from each cross several lines were derived, genomic predictions can have a greater correlation to the true unknown genotypic values than the phenotypic values. Hence, genomic selection has the potential to increase the efficiency of breeding programs that use low-intensity field trials.

## Data Availability Statement

Simulated data are available from the authors on request. Requests to access these datasets should be directed to biometry.popgen@uni-giessen.de. The R code used for the simulations is available at https://github.com/JT-Giessen/Terraillon_2022.

## Author Contributions

MF and FO conceived the study. HJ, MS, LC, KK, SB, AH, DK, and AS collected the data of the barley experiment. KCF and CZ-P analyzed the data of the barley experiment. JT and MF carried out the simulations. JT, CZ-P, and MF wrote the manuscript. All authors read and approved the final manuscript.

## Funding

The project was supported by funds of the Federal Ministry of Food and Agriculture (BMEL) based on a decision of the Parliament of the Federal Republic of Germany *via* the Federal Office for Agriculture and Food (BLE) under the innovation support programme (FKZ 2818203515).

## Conflict of Interest

HJ was employed by Saatzucht Josef Breun GmbH & Co. KG. MS was employed by KWS Lochow GmbH. LC was employed by W. von Borries-Eckendorf GmbH & Co. KG. KK was employed by Limagrain GmbH. SB was employed by Ackermann Saatzucht GmbH & Co. KG. The remaining authors declare that the research was conducted in the absence of any commercial or financial relationships that could be construed as a potential conflict of interest.

## Publisher's Note

All claims expressed in this article are solely those of the authors and do not necessarily represent those of their affiliated organizations, or those of the publisher, the editors and the reviewers. Any product that may be evaluated in this article, or claim that may be made by its manufacturer, is not guaranteed or endorsed by the publisher.
